# Functional motifs in *Escherichia coli* NC101

**Published:** 2013

**Authors:** Gholamreza Motalleb

**Affiliations:** Department of Biology, University of Zabol, Zabol, Iran.

**Keywords:** *Escherichia coli*, functional motifs, gene expression

## Abstract

*Escherichia coli* (*E. coli*) bacteria can damage DNA of the gut lining cells and may encourage the development of colon cancer according to recent reports. Genetic switches are specific sequence motifs and many of them are drug targets. It is interesting to know motifs and their location in sequences. At the present study, Gibbs sampler algorithm was used in order to predict and find functional motifs in *E. coli *NC101 contig 1. The whole genomic sequence of *Escherichia coli* NC101 contig 1 were retrieved from http://www.ncbi.nlm.nih.gov (NCBI Reference sequence: NZ_AEFA01000001.1) in order to be analyzed with DAMBE software and BLAST. The results showed that the 6-mer motif is CUGGAA in most sequences (genes1-3, 8, 9, 12, 14-18, 20-23, 25, 27, 29, 31-34), CUUGUA for gene 4 , CUGUAA for gene 5, CUGAUG for gene 6, CUGAUA for gene7, CUGAAA for genes 10, 11, 13, 26, 28, and CUGGAG for gene 19, and CUGGUA for gene30 in E. coli NC101 contig 1. It is concluded that the 6-mer motif is CUGGAA in most sequences in E. coli NC101 contig1. The present study may help experimental studies on elucidating the pharmacological and phylogenic functions of the motifs in *E. coli*.

Infection of eukaryotic cells with pks+ *E. coli* strains induces host-cell DNA double strand breaks (DSBs) and activation of the DNA damage signaling cascade, including the ATM–CHK–CDC25–CDK1 pathway and Ser139 phospho-rylation of histone H2AX (1). A genome can assure cells life whenever its encoded genes are activated or inactivated during molecular and cellular changes in order to answer to environmental factors and production of various RNA and proteins on time and correct place (2). The aim of motif discovery is to find patterns in protein or nucleotide sequences to understand the function and structure of the molecules the sequences represent (3). Motif extraction of MLA or multiple local alignments is often used to determine DNA sites that are distinguished by TF or transcription factors. This is based on the assumption that DNA sequences upstream of coregulated genes contain similar nucleotide subsequences (4). Genetic switches are specific sequence motifs and many of them are drug targets (5). These contain intron branching-point site, transcription factor binding sites, intron-splicing sites, etc. Gibbs sampler is a Monte Carlo algorithm used in order to find these motifs (5). Monte Carlo algorithm method was created by Stanislaw Ulam and developed by nuclear weapon projects in USA (6). Gibbs sampler has been used to identify functional motifs in proteins (7), multiple sequence alignment (8), and biological image processing (9). The main element of a Gibbs sampler is position weight matrix or PWM. The PWM scores or PWMS has been reported as a scale of the motif strength (5). *Escherichia coli* are anaerobic bacteria and the most common population of bacteria in the intestinal flora of human. *E. coli* can make colony in the intestine few days after birth and permanently during human life. Strains of *E. coli* can be categorized into four main groups (A, B1, B2, and D) and B2 group can persist in the colon longer than the others (10). It was reported that *E. coli* strains of B2 phylotype (e.g. *E. coli* NC101), carry a genomic *pks* island (a gene cluster coding nonribosomal peptide synthetases or NRPS and polyketide synthetases or PKS), produce Colibactin (a peptide-polyketide genotoxin) that can induce damage of DNA by double-strand breaks (DSBs) (11) and may develop colon cancer (12). In the present study, Gibbs sampler was used to identify functional motifs by BLAST and DAMBE software in order to distinguish motifs in *E. coli* strain NC101 contig1.

## Materials and Methods

This investigation was started in the spring of 2013 and the data analysis was performed at bioinformatics facility of Faculty of Science at Zabol University. Genome sequences of *E. coli* NC101 (NCBI Reference sequence: Z_AEFA01000001.1) were retrieved from http://www.ncbi.nlm.nih.gov (NCBI Reference sequence: NZ_AEFA01000001.1) to find branch point sequence or BPS in *E. coli* NC101 contig1 by DAMBE (5). 

BLAST search of the *E. coli* NC101 genome (accession NZ_AEFA00000000) confirmed the presence of pks. PWM is computed as ${\mathrm{PWM}}_{\mathrm{ij}}={\mathrm{Log}}_{2}\left(\frac{p_{\mathrm{ij}}}{p_{i}}\right)$           (1).

Where i=1, 2, 3 and 4 corresponding to A, C, G and U, respectively, and j is site index, and *p*_i _is the background frequency of nucleotide *i*, and *p*_ij_ is the site specific nucleotide frequency for nucleotide *i *at site *j*. The PWMS for a particular motif is computed as $\mathrm{PWMS}=\sum_{j=1}^{L}{\mathrm{PWM}}_{\mathrm{i.j}}$ (2) where L is length of the motif (5).

## Results

**Fig 1:**
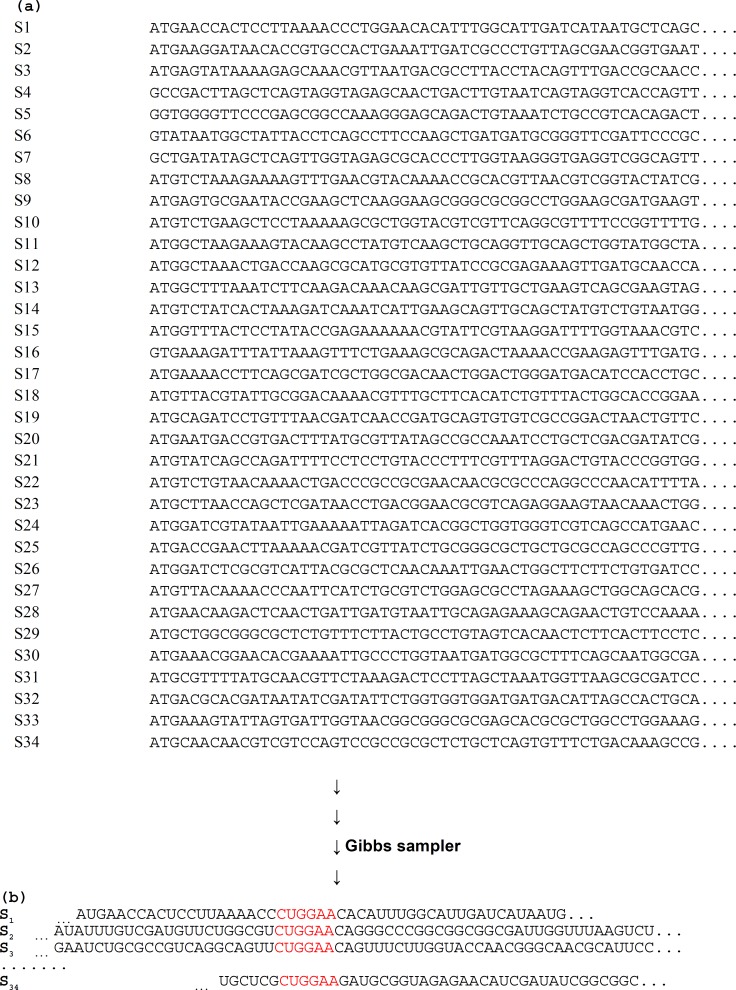
The sequences of *E. coli *NC101 contig1. The above panel represents the data input in Gibbs sampler (a). The below part represents the output of the motifs (i.e.,CUGGAA; in red color) through the sequences (b). S1-S34 correspond to sequence 1 to sequence 34

**Fig. 2:**
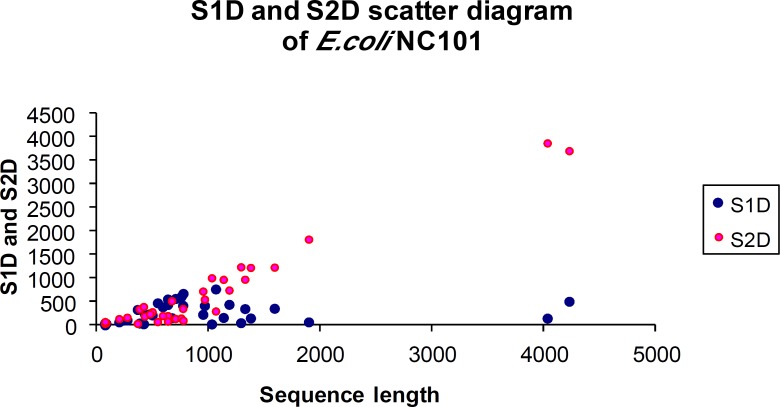
Scatter diagram of S1D and S2D in *E. coli* NC101 contig1 sequences

Gibbs sampler was employed to find functional motifs by DAMBE in order to identify genetic motifs with Gibbs sampler in *E. coli* NC101 contig1. Figure 1 shows shared motif in an aligned format in red color which is CUGGAA in most sequences (Fig. 1b). The main Gibbs sampler output is the sequences with aligned motifs as shown in Figure 1b and a site-specific frequency matrix (position weight matrix) presented in Table 1d respectively. Table 1a shows the total number of nucleotides in the sequences. The total number of nucleotides for 34 sequences is 31509, with 7622, 7977, 8879 and 7031 for A, C, G and U respectively. The partial output (Table 1b and c) showed that the 6-mer motif is CUGGAA. The site-specific frequencies and PWM were shown in Table 1c and d in order to find and monitor other sequences for the presence of such motifs. The last part of the results (Table 2) shows the motifs start point. As shown again in Table 2, the 6-mer motif is CUGGAA in most sequences. Figure 2 shows the scatter diagram of S1D and S2D with *E. coli* NC101 contig1 sequences length**.**

## Discussion

The finding of motifs in DNA sequences is a central problem in computational molecular biology, and through many computational methods, Gibbs sampling algorithm is a great promise which is used for finding functional motifs in the co-expressed genes (13). Motif finding is becoming an important toolbox for microbiologists likewise other DNA and protein computational molecular

**Table 1:** Gibbs sampler output

**(a)** Global alignment score (F) = 230.9101 Frequency Table				
Code	Count	Freq		
A	7622	0.2419		
C	7977	0.2532		
G	8879	0.2818		
U	7031	0.2231		
**(b)** Final site-specific counts				
	A	C	G	U
1	0	34	0	0
2	0	0	0	34
3	0	0	33	1
4	7	0	26	1
5	29	0	0	5
6	32	0	2	0
**(c)** Final site-specific frequencies				
	A	C	G	U
1	0.00691	0.97866	0.00805	0.00638
2	0.00691	0.00723	0.00805	0.97780
3	0.00691	0.00723	095091	0.03495
4	020691	0.00723	0.75091	0.03495
5	0.83548	0.00723	0.00805	0.14923
6	0.92120	0.00723	0.06519	0.00638
**(d)** Final PWM				
	A	C	G	U
1	3.55288-	1.34992	3.55495-	3.55600-
2	3.55288-	3.55757-	3.55495-	1.47685
3	3.55288-	3.55757-	1.21665	1.85463-
4	0.15376-	3.55757-	0.98051	1.85463-
5	1.24196	3.55757-	3.55495-	0.40295-
6	1.33962	3.55757-	1.46340-	3.55600-

Number of input sequences: 34; Width of motif: 6. a: Frequency table; b: Final site-specific counts in motifs; c: Final site-specific frequencies in motifs; d: Final PWM in motifs. A= adenine; C= cytosine; G= guanine; U= uracil biology sequence analysis methods. These techniques can provide very useful and valuable information with very lower cost compared to laboratory experiments. The most common application of motif finding is to determine and find the TFBS or transcription factor binding sites (14). Transcription factors (TFs) attach most often to small segments of DNA (binding sites) in DNA upstream of a gene to activate or inactivate of gene transcription. Their DNA-binding domains can distinguish and recognize motifs. TRPF or transcription regulatory protein factors often connect to DNA as homo or hetero-dimers. Therefore they distinguish DNA motifs that are spaced motif pairs, inverted or direct repeats. However, these motifs are often tedious and difficult to identify owing to their high divergence (15). Because of multiple binding modes and indirect recognition, the action and reaction of operators and holorepressors are highly sensitive to the experimental conditions (DNA length, buffer components, etc). Thus, there are many discrepancies about equilibrium constants, kinetic data and stoichiometry for these complexes. In other words, equilibrium binding constants for holorepressor/operator is different from one experiment to another (15-16). However, there is no unique combination of bases that is shared by all binding sites, and although different bases can occur at each position, there are clear biases in the distribution of bases that occur at each position of the binding sites (17-18). PWM has been employed in genome investigations such as whole genome identification of transcription units (19), transcription factor binding sites or TFBS (20), transcription initiation sites (21) and translation initiation sites (22). Position weight matrix sequence analysis has three outputs: the site specific frequency, the position weight matrix, and PWMS. On the other hand, it is interesting to find sequence motifs in a set of co expressed genes by microarray experiments (23). If these genes are co regulated, thus they share TFBS that could be monitored or controlled by similar or common TF (24). Gibbs sampler will output a quantitative measure of the motifs by computer program. PWMS is the log-odds ratio, and the strongest motif has the highest PWMS or odds-ratio (5). In this work, Gibbs sampler algorithm was employed to find the functional motifs in *E. coli* NC101 contig1 sequences. The results showed that CUGGAA is a 6-mer motif that has the highest PWMS of 2009.2156. 24 out of 34 sequences genes, had the 6-mer motif of CUGGAA (70.58%) (Table 2). The homology between genes based on sequence motifs is very important and crucial in order to understand the function of uncharacterized genes and may be helpful in studying the dynamic behavior of genes (25). That is, it may be concluded that they may be co regulated. Recently, the researchers reported that the transfer of a functional gene from bacteria to mammalian cells could occur. They showed that engineered *E. coli*, expressing *Inv* and *HlyA *genes (from *Yersinia pseudotuberculosis* and *Lysteria monocytogenes*, respectively) are able to attack and release DNA into mammalian cells (26). The similar phenomenon was also reported *in vivo*, and it was shown that invasive *E. coli* can carry and deliver therapeutic genes to the colonic mucosa in mice (27). On the other hand, a successful shRNA transfer into mammalian cells was carried out by non-pathogenic *E. coli* through a plasmid (28). Bacteria strains for example *E. coli*, Salmonella, and Clostridium can selectively grow and colonize in tumors. In fact, scientists have showed that bacteria are able to attack primary tumors and metastases and they can be used for tumor-selective drug delivery (29). 

**Table 2:** Gibbs sampler results of *E. coli* NC101 contig1 sequences for motif, start location and PWMS identification

SeqName	Motif	Start	PWMS
lcl|NZ_AEFA01000001.1_gene_1	CUGGAA	20	2009.2156
lcl|NZ_AEFA01000001.1_gene_2	CUGGAA	414	2009.2156
lcl|NZ_AEFA01000001.1_gene_3	CUGGAA	225	2009.2156
lcl|NZ_AEFA01000001.1_gene_4	CUUGUA	31	17.9811
lcl|NZ_AEFA01000001.1_gene_5	CUGUAA	32	117.9619
lcl|NZ_AEFA01000001.1_gene_6	CUGAUG	39	7.5632
lcl|NZ_AEFA01000001.1_gene_7	CUGAUA	1	124.7508
lcl|NZ_AEFA01000001.1_gene_8	CUGGAA	438	2009.2156
lcl|NZ_AEFA01000001.1_gene_9	CUGGAA	39	2009.2156
lcl|NZ_AEFA01000001.1_gene_10	CUGAAA	471	646.2746
lcl|NZ_AEFA01000001.1_gene_11	CUGAAA	237	646.2746
lcl|NZ_AEFA01000001.1_gene_12	CUGGAA	564	2009.2159
lcl|NZ_AEFA01000001.1_gene_13	CUGAAA	213	646.2746
lcl|NZ_AEFA01000001.1_gene_14	CUGGAA	330	2009.2156
lcl|NZ_AEFA01000001.1_gene_15	CUGGAA	144	2009.2156
lcl|NZ_AEFA01000001.1_gene_16	CUGGAA	504	2009.2156
lcl|NZ_AEFA01000001.1_gene_17	CUGGAA	159	2009.2159
lcl|NZ_AEFA01000001.1_gene_18	CUGGAA	417	2009.2156
lcl|NZ_AEFA01000001.1_gene_19	CUGGAG	63	121.8117
lcl|NZ_AEFA01000001.1_gene_20	CUGGAA	606	2009.2156
lcl|NZ_AEFA01000001.1_gene_21	CUGGAA	435	2009.2156
lcl|NZ_AEFA01000001.1_gene_22	CUGGAA	63	2009.2156
lcl|NZ_AEFA01000001.1_gene_23	CUGGAA	237	2009.2156
lcl|NZ_AEFA01000001.1_gene_24	CUGGUA	671	387.8401
lcl|NZ_AEFA01000001.1_gene_25	CUGGAA	765	2009.2156
lcl|NZ_AEFA01000001.1_gene_26	CUGAAA	153	646.2746
lcl|NZ_AEFA01000001.1_gene_27	CUGGAA	387	2009.2156
lcl|NZ_AEFA01000001.1_gene_28	CUGAAA	105	646.2746
lcl|NZ_AEFA01000001.1_gene_29	CUGGAA	552	2009.2156
lcl|NZ_AEFA01000001.1_gene_30	CUGGUA	24	387.8401
lcl|NZ_AEFA01000001.1_gene_31	CUGGAA	147	2009.2156
lcl|NZ_AEFA01000001.1_gene_32	CUGGAA	348	2009.2156
lcl|NZ_AEFA01000001.1_gene_33	CUGGAA	47	2009.2156
lcl|NZ_AEFA01000001.1_gene_34	CUGGAA	354	2009.2156

Mean                                         1429.4078

Standard deviation                    812.3610

Our results may help the mentioned scenario by finding and discovering the functional genetic switches and motifs in *E. coli* NC101 contig1. The branch point sequence could be placed anywhere, however, it is preferable to be near the 3’ rather than the 5’ site. Surely, experiments causing step by step mutation on each nucleotide of the sequence between the donor and the acceptor site, could be performed, but this is very tedious and difficult. Therefore, one can apply and run the Gibbs sampler in order to find all the BPSs. The BPS cuts the *E. coli* NC101 contig1 sequences into two sections: the upstream part stretching from the 5’ site to BPS (the S1 sequence), and the downstream sequence from BPS to the 3’ site (the S2 sequence). The lengths of S1 and S2 sequences are named as S1 and S2 distances (S1D and S2D). If BPS is limited to be near the 3’ site, thus the S2 distance is smaller than the S1 distance and vice versa (5). 

Scatter diagram of S1D and S2D of *E. coli* NC101 contig1 sequences is shown in Figure 2. The results showed that most of the S2D were higher than S1D (650.11±157.24 and 270.61±37.17 respectively).

The present study may help experimental studies on elucidating the pharmacological and phylogenic functions of the motifs in *E. coli*.

## Conflict of interest

Authors declared no conflict of interest.
